# A Comparison of Three-Dimensional Speckle Tracking Echocardiography Parameters in Predicting Left Ventricular Remodeling

**DOI:** 10.1155/2020/8847144

**Published:** 2020-07-30

**Authors:** Junda Zhong, Peng Liu, Shuang Li, Xiaomin Huang, Qunhui Zhang, Jianyu Huang, Yan Guo, Meixiang Chen, Zheng Ruan, Changyu Qin, Lin Xu

**Affiliations:** ^1^Department of Geriatric Cardiology, General Hospital of the Southern Theatre Command, PLA, Guangzhou 510016, China; ^2^The First School of Clinical Medicine, Southern Medical University, Guangzhou 510515, China; ^3^Department of Cardiology, Zhujiang Hospital, Southern Medical University, Guangzhou, China; ^4^The Second School of Clinical Medicine, Southern Medical University, Guangzhou 510280, China

## Abstract

Three-dimensional speckle tracking echocardiography (3D STE) is an emerging noninvasive method for predicting left ventricular remodeling (LVR) after acute myocardial infarction (AMI). Previous studies analyzed the predictive value of 3D STE with traditional models. However, no models that contain comprehensive risk factors were assessed, and there are limited data on the comparison of different 3D STE parameters. In this study, we sought to build a machine learning model for predicting LVR in AMI patients after effective percutaneous coronary intervention (PCI) that contains the majority of the clinical risk factors and compare 3D STE parameters values for LVR prediction. We enrolled 135 first-onset AMI patients (120 males, mean age 54 ± 9 years). All patients went through a 3D STE and a traditional transthoracic echocardiography 24 hours after reperfusion. A second echocardiography was repeated at the three-month follow-up to detect LVR (defined as a 20 percent increase in left ventricular end-diastolic volume). Six models were constructed using 15 risk factors. A receiver operator characteristic curve and four performance measurements were used as evaluation methods. Feature importance was used to compare 3D STE parameters. 26 patients (19.3%) had LVR. Our evaluation showed that RF can best predict LVR with the best AUC of 0.96. 3D GLS was the most valuable 3D STE parameters, followed by GCS, global area strain, and global radial strain (feature importance 0.146, 0.089, 0.087, and 0.069, respectively). To sum up, RF models can accurately predict the LVR after AMI, and 3D GLS was the best 3D STE parameters in predicting the LVR.

## 1. Introduction

Acute myocardial infarction (AMI) has been the leading cause of cardiac death among all the cardiovascular events. According to the China cardiovascular disease report 2018 [1], the prevalence of AMI in Chinese urban area is 0.999‰, which is still growing, with a total number of 753,142 percutaneous coronary interventions (PCIs) being carried out in 2017. Left ventricular remodeling (LVR), an structural adaptation of the myocardium to compensate for the contractile dysfunction of myocardial fibers [2], is an important reference in early cardiac rehabilitation treatment for it is the main cause of heart failure after AMI [3–6]. Therefore, a robust prediction on the occurrence of LVR is invaluable to the recovery of AMI patients.

Some studies have explored the values of serological indicators, echocardiographic parameters, cardiac magnetic resonance imaging (CMRI) [7–10], and coronary angiography (CAG) in LVR prediction. Among all the imaging examinations, echocardiography is most vastly applied because it is less costly, less time-consuming, and friendly to almost all types of patients, with a good balance of simplicity and predictive power. Three-dimensional speckle tracking echocardiography (3D STE) is a noninvasive method that outmatches traditional echocardiography in diagnostic power and other imaging methods both in an economical or a practical term [10–12]. 3D STE tracks the deformation of the myocardium through actual three-dimensional observations rather than geometrical assumptions. There have been some studies that used 3D STE to predict LVR [13–16], but few studies have compared the abilities of different 3D STE parameters in predicting LVR or built a comprehensive model to predict LVR using factors that include 3D STE.

With the growing attention on machine learning, the medical application of this technology has become a new focus [17]. Machine learning is an interdisciplinary subject, which involves probability theory, statistics, approximation theory, convex analysis, algorithm complexity theory, and other disciplines [18–22]. It is flexible, expandable, and automatic, which makes it adaptable for risk stratification, diagnosis, and predictions, but currently, we cannot find any machine learning algorithm being applied to predict the occurrence of LVR.

In this study, we attempted (1) to investigate the prediction power of machine learning methods in predicting LVR and (2) to investigate the difference of 3D STE parameters in predicting LVR.

## 2. Materials and Methods

### 2.1. Patient Population and Protocols

172 consecutive patients with first-onset AMI were initially enrolled in this study. All the AMI patients were diagnosed according to the guideline recommendations. Exclusion criteria were as follows: age <18 years, a history of previous coronary heart disease requiring a PCI, severe valvulopathy, left bundle branch block, atrial fibrillation, malignant arrhythmia, and/or any condition compromising the patient's ability to comply. Patients received reperfusion within 12 hours. 24 h after effective PCI, patients went through a standard transthoracic echocardiography and a 3D STE examination. After three months, patients went through another standard transthoracic echocardiography. We defined LVR as an increase ≥20% in LVEDV at three-month follow-up [2, 23–28]. The study protocol was approved by the ethics committee of the General Hospital of the Southern Theatre Command, PLA, and oral informed consent was obtained from all the patients. Due to the sensitivity of patients' personal information in a military hospital, an application for waiver of written informed consent was applied and approved by the same ethics committee (No. 202041).

### 2.2. 3D STE Examinations

**Figure 1:**
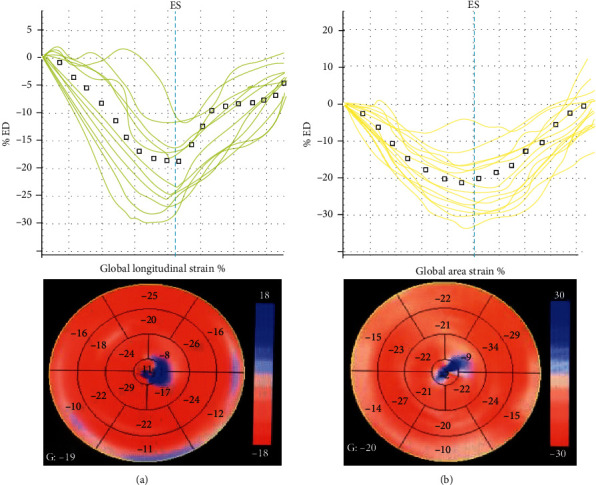
Three-dimensional speckle tracking echocardiography (3D STE) analysis shows the result of global longitudinal strain (GLS) and global area strain (GAS) on a bull's eye depiction acquired by EchoPAC 112 (GE Medical System, Horten, Norway) from a patient. (a) Curves of instantaneous segmental 3D GLS in a patient (−18.9%). (b) Curves of instantaneous segmental 3D GAS in a patient (−21.8%).

3D STE was performed using a GE Vivid E9 ultrasound diagnostic system (Horten, Norway) with a 4D volume probe (4 V-D). First, left ventricular volume data from an apical four-chamber view of four to six consecutive ECG-gated cardiac cycles were obtained and stored during a single end-expiratory breath hold. Then, we outlined the LV endocardial and epicardial borders as a region of interest. Then, the 3D GLS, 3D GCS, 3D GRS, and 3D GAS values were displayed in a bulls-eyed plot (Figure 1).

According to Korup et al., left ventricular dilatation began within three hours after acute myocardial infarction, and no further progress was made after that in the first six days [29]. Sakuma et al. reported that the optimal timing to detect myocardial changes for predicting LVR is 24 hours after reperfusion of the culprit artery [30]. Based on these studies, we assessed 3D myocardial contractions at 24 hours after PCI.

### 2.3. Coronary Angiography

All patients went through CAG to identify an infarct-related artery (IRA), measure the thrombolysis in myocardial infarction (TIMI) grade, and carry out revascularization through PCI. CAG was performed with a digital subtraction angiography machine. For coronary artery reperfusion therapy, subsequent PCI was performed to recover blood flow in the IRA. The blood flow level of the coronary artery was measured with the TIMI grade during CAG both at baseline and after coronary angioplasty. Patients with a TIMI grade ≥3 after coronary angioplasty were included in the statistical analysis.

### 2.4. Statistics

All statistical analyses were performed using IBM SPSS 21.0 (Chicago, IL, USA) software and Python with modules including Scikit-learn based on Abraham A's method [31], as well as Pandas, Numpy, Tensorflow, and Matplotlib. Data as continuous variables were expressed as means ± SD. Categorical variables were presented as absolute numbers and relative frequencies. Normal distribution of variables was checked with the Kolmogorov–Smirnov test. Continuous variables were compared using Student's *t*-test. Fisher's exact test or the chi-squared test was used to compare categorical variables.

In this study, before we compared 3D STE in a specific model, we used 15 risk factors to build six models including Decision Tree (DT), Random Forest (RF), eXtreme Gradient Boosting (eXGB), K-Nearest Neighbors (K-NN), Gaussian Naive Bayes (GNB), and Logistic Regression (LR) and compared the prediction power of all six models. The modules of the above machine learning methods were imported into Python so that no extra coding was needed. 5-fold cross-validation was performed to enhance the effect of testing and modeling capability. A receiver operator characteristic (ROC) curve was performed, and area under the curve (AUC), accuracy, sensitivity, specificity, and F1 score were calculated to evaluate classifiers.

**Figure 2:**
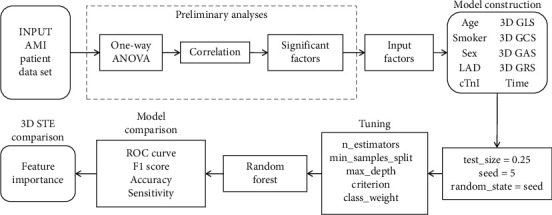
Data analysis work flow.

In this study, data analysis proceeded according to the following steps. (1) Preliminary analysis: input patient data set and conduct one-way ANOVA, chi-square, and correlation analysis. (2) Model construction: input significant factors from step one, and import modules including DT, RF, eXGB, K-NN, GNB, and LR to Python. Each parameter was tested under 5-fold cross-validation that randomly selected 75% of the dataset as the training set and the rest 25% as the test set. (3) Tuning: conducted multiple program running and sorted out the best values of the model parameters such as n_estimators, max_depth, and random_state. (4) Model comparison: compared constructed models using AUC, accuracy, sensitivity, specificity, and F1 score. (5) 3D STE comparison: after the best classification method was confirmed, we compared 3D STE parameters through feature importance from the model. The data analysis work flow is displayed in Figure 2.

## 3. Results

### 3.1. Demographic and Clinical Characteristics

Initially, there were 172 patients enrolled in this study. 37 were further excluded due to the following reasons: (1) 13 patients with a TIMI grade < 3, (2) 12 patients due to poor myocardial tracking (>2 nonvisualized segments), (3) 10 patients for disagreement to participate, and (4) 2 patients died. Eventually, 135 patients (mean age, 54 ± 9, 88.9% males) were included in our study.

Patients were divided into two groups according to the occurrence of LVR.  Table 1 displays baseline demographic and clinical characteristics. Age, sex, body mass index, body surface area, medical history, angiographic findings, blood tests findings as well as medication during follow-up were compared. Patients with LVR were older than patients without LVR (56.85 ± 11.80 yrs vs. 53.22 ± 7.92 yrs, *p* = 0.044, S). There is no significant difference among the other characteristics.

### 3.2. Echocardiographic Data

Baseline and three-month follow-up standard echocardiographic parameters as well as baseline 3D STE parameters are presented in Table 2. 26 patients (19.3%) were defined as LVR (>20% increase in LVEDV), and 109 patients (80.7%) did not have LVR. No significant differences were found in baseline standard echocardiographic characteristics between LVR and non-LVR patients. Follow-up LVEDV, LVESV, and LVEF were all significantly different between the two groups (respectively, 126.94 ± 19.77 vs. 105.32 ± 25.53, *p* < 0.001; 62.39 ± 14.12 vs. 47.86 ± 18.34, *p* < 0.001; 51.43 ± 7.00 vs. 55.42 ± 8.79, *p* = 0.025). Follow-up LVMI was not significant between the LVR and non-LVR patients.

A 3D STE assessment was carried out 24 hours after effective PCI (defined as a TIMI grade ≥ 3). The results are also presented in Table 2. 3D GLS and 3D GRS in patients with LVR were significantly reduced (respectively, −9.90 ± 2.60% vs. −12.99 ± 3.10%, *p* < 0.001 and 28.13 ± 7.13% vs. 32.29 ± 9.43%, *p* = 0.037).

### 3.3. LVR Risk Factors

A correlation analysis was conducted to find out possible risk factors that have impact in predicting LVR. The results are presented in Table 3. Age, 3D GLS, 3D GCS, and 3D GRS were correlated with the occurrence of LVR. Among all of the 3D STE parameters, the *r* value of 3D GLS is the best, the second is 3D GRS, and the third is 3D GCS. In this correlation analysis, 3D GAS does not correlate with the occurrence of LVR.

Univariate analysis showed that 3D GLS, 3D GRS, and LVMI were associated with LVR occurrence. The odds ratio (OR) and 95% CI for each of 3D STE parameters along with other factors are displayed in Table 4. One of the important findings in the univariate analysis was that 3D GLS was the best predictor of LVR occurrence (OR, 1.374; 95% CI, 1.176–1.604; *p* < 0.001). And 3D GRS was also a good predictor (OR, 0.949; 95% CI, 0.903–0.998; *p* = 0.040). Further assessment of these factors was conducted by using machine learning methods to build models that contained most of the clinically important factors.

### 3.4. LVR Predictive Models

DT, RF, eXGB, K-NN, GNB, and LR were applied to construct models with 15 clinical risk factors including age, sex, smoking, BMI, body surface area, serum creatinine, cTnL, time to perfusion, left anterior descending branch occlusion as the infarct-related artery, multivessel occlusion, LVMI, and four 3D STE parameters. The constructed models were then compared to show which was the best in predicting LVR in this sample.

**Figure 3:**
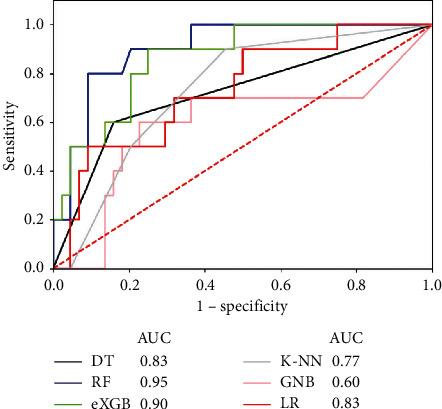
Comparison of the ROC curves of all models. The Random Forest model showed the best AUC of 0.96 (blue line), and eXGB showed the second best AUC of 0.90 (green line).

Merged ROC curves of all six classifiers are presented in Figure 3.  Table 5 shows all the evaluation parameters of the constructed models. As a result, the RF model predicted LVR with the best AUC of 0.96, the best accuracy of 90.48%, and the second best specificity of 94.12%, surpassing the other models. eXGB ranked second to RF with an AUC of 0.90. DT and LR ranked third with equal AUCs of 0.83. The K-NN model had an AUC of 0.77, and GNB had the lowest AUC of 0.60. LR and K-NN had the best sensitivity (94.64% and 92.86%).

**Figure 4:**
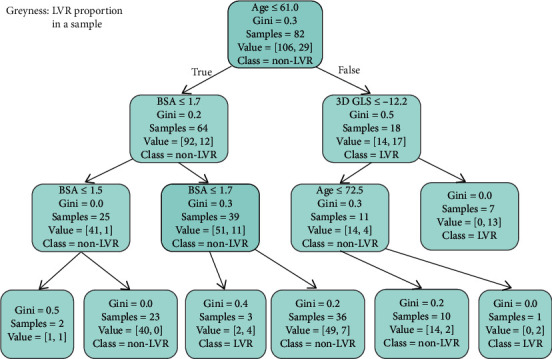
One of the decision trees in the resulted Random Forest model. A Random Forest model is a combination of multiple diverse decision trees. The decision tree displayed in this figure had age, 3D GLS, and BSA as classification features. The grayness in a box means the probability of a node being predicted as LVR. The parameter “sample” means the number of randomly chosen samples in this node. The parameter “Gini” measures the diversity of the samples, that is, the probability of inconsistent categories between two samples from a data set. The smaller the Gini index, the higher the purity of the sample. The parameter “class” means this node tendency of this vote.

Since the RF model was the best in this work, we further ran a visualization of the model structure. The structure of one of the decision trees that formed the RF model is visualized and displayed in Figure 4. For each sample, a decision tree identifies it through multiple nodes and finally contributes a vote to decide if it is LVR or non-LVR. Each decision tree might have different features and different number of nodes. In the example given in Figure 4, the decision tree votes its decision through three processes: first, the value of a sample's age; second, the BSA or 3D GLS; and final, the BSA or age.

### 3.5. Comparison of Different 3D STE Parameters in Predicting LVR

**Figure 5:**
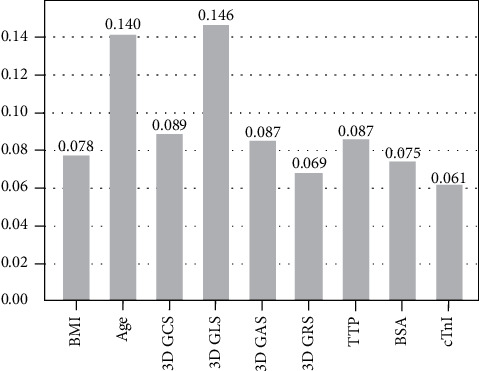
Feature importance in the Random Forest model. It showed that among all the 3D STE parameters, 3D GLS was the most important one, with an importance of 0.146, followed by 3D GCS with an importance of 0.089, 3D GAS with an importance of 0.087, and 3D GRS with an importance of 0.069.

As a result of the above section, we found that RF can construct the best model to predict LVR, and consequently, we used such model to display the comparison of different 3D STE parameters' ability in predicting LVR. The model was trained under 5-fold cross-validation that randomly selected 75% of the sample as the training set (*n* = 101) and 25% of the sample as the test set. A feature importance analysis of the RF model was conducted, and the resulted diagram is displayed in Figure 5. The five most important features of the RF model were 3D GLS, age, 3D GCS, time to perfusion (TTP), and 3D GAS (feature importance: 0.146, 0.140, 0.089, 0.087, 0.087, respectively).

## 4. Discussion

It is difficult to predict which AMI patients will and which will not develop LVR after a successful PCI. We built several prediction models including the conventional model and machine learning models and discovered that RF achieved higher predictive power than other models in our work and used the Random Forest model to compare 3D STE parameters, finally discovering the overwhelming predictive value of 3D GLS, thus bringing more attention to possible future investigation into 3D GLS. Our study was the first to build a machine learning model for LVR prediction using factors that were mostly encountered in clinical practice plus four 3D STE parameters and compare 3D STE parameters values for predicting LVR in AMI patients after effective PCI by using the Random Forest method.

### 4.1. Predictive Models for LVR

In this study, we built a strong RF model for LVR prediction, using most of the important factors we encountered in the clinical practices. Some studies also built various predictive models for LVR in AMI patients. Bochenek et al. built a regression model using global longitudinal strain solely as a risk factor, with an AUC of 0.77 and accuracy of 80% [32]. Sugano et al. used 3D GCS to predict LVR, with an AUC of 0.73 and sensitivity of 84% [33]. Xu et al. built regression models that contained several clinical risk factors, but their work focused on evaluating these factors and did not assess these models' ability as a whole in predicting LVR [34]. Most of the studies build regression models to investigate the predictive value of a separate risk factor. We did not find studies that assessed models using various clinical risk factors, whether it included 3D STE parameters or not.

### 4.2. The Predictive Value of 3D STE for LVR

This study demonstrated that 3D GLS, among all the 3D STE parameters, is the strongest in predicting LVR in AMI patients undergoing effective PCI, the power of which exceeded other conventional markers such as cTnI, which is consistent with most similar studies [15, 32, 35–37]. We assumed that this phenomenon partly resulted from the intuitive feature of 3D STE in observing heart movement. The effect of cTnI on the occurrence of LVR is subtle, and the same goes for other serological biomarkers, while 3D STE detects detailed heart movement to predict probable myocardial changes in the future.

In our study, due to its original characteristics, the RF model constructed requires less calculation and fits better in real-world clinical cases, in which the samples are usually small and imbalanced.

### 4.3. Different 3D STE Parameters in Predicting LVR

In our model generated by RF, 3D GLS was the most important feature, as shown in Figure 5 (feature importance of 3D GLS: 0.146, age: 0.140, 3D GCS: 0.089, TTP: 0.087, and 3D GAS: 0.087). This result is in consistence with many other studies that used traditional biostatistical models. A reasonable explanation for the excellent performance of 3D GLS lies in the anatomic characteristic of the coronary artery and the capillary network inside the heart muscles. 3D GLS observes the most vulnerable myocardium layer, the sub-endocardium, which is anatomically far from the coronary artery and receives the least nutrition from its capillary network, rendering it the most vulnerable to coronary artery blockage. These myofibrils are the first to show abnormality in a heart attack and remain poorly cared for after the revascularization, in which the affected endocardial movement is uncoordinated, and the amplitude is reduced.

In one of our previous studies, we compared 3D STE parameters with 2D STE in predicting LVR in ST-elevated myocardial infarction patients, coming to the conclusion that 3D GRS was the second best 3D STE parameter, followed by 3D GAS, while 3D GCS showed no predictive effect [36]. However, in this study, we had a different result. 3D GCS was the second best 3D STE parameter, followed closely by 3D GAS (feature importance 0.089 and 0.087). As Random Forest commonly has a better result in small and imbalanced sample and we included more risk factors in this work, we believe this result is more accurate, but further investigation is needed to confirm this theory.

### 4.4. Random Forest Model

In this work, we decided to compare the 3D STE parameters with the RF model for it had the best performance, outmatching DT, eXGB, K-NN, GNB, and LR models. The DT is simple in calculation and vastly used, but overfitting remains as one of its main disadvantages, which might be the reason why it did not have a good performance in this work. The eXGB is a highly efficient and optimized distributed gradient boosting library [38]. It is highly flexible and portable, which excels in big data analysis. However, the imbalance of a dataset can affect the training of an eXGB model, which explains why it was not as good as RF in this work. The K-NN algorithm searches the most similar training samples to predict the observation value of a new sample. It usually performs well in numerical data and discrete data, but performs badly when the sample is imbalanced, which is quite opposite to the RF. The Gaussian NB usually has a good performance in small sample studies, but in this study, it still performed badly. We assumed the main reason was that many of the variables in this study were discontinuous, which may affect the power of Gaussian NB. The other reason is that the Gaussian NB presumed that none of the variables interact with each other, which is unlikely in this study, and this may heavily affect the predictive power of the Gaussian NB model, and the LR performed badly in this work for the same reason.

Random Forest is a highly flexible machine learning algorithm that performs well in small and imbalanced samples [19]. That explains why it excelled in this study. It is based on bagged decision trees that are trained on bootstrap samples. And these decision trees combined and formed a Random Forest. Its coding was uploaded in the supplement files.

In the our RF model, Gini impurity was used to measure the partitioning attribute. Assuming that the proportion of the *k*^th^ sample in the current sample set *D* is *p*_*k*_ (*k* = 1, 2,…, *K*), the purity of the dataset *D* can be measured by the Gini value:

(1) $$\begin{array}{c} \text{Gini}\left(D\right)=\sum_{k=1}^{K}\sum_{k^{\prime }\neq k}p_{k}p_{k^{\prime }}=1-\sum_{k=1}^{K}p_{k}^{2}. \end{array}$$

When Gini (*D*) = 0, the sample was the purest, and then, the category extracted was of the same type, either LVR or non-LVR. When Gini (*D*) = 0.5, the probabilities of two categories were the same, meaning the tree cannot distinguish LVR or non-LVR. Therefore, the smaller the Gini (*D*), the higher the purity of the dataset *D*. For the tuning of parameters, see the supplementary materials (available here).

An RF can be described as a cluster of many decision trees in which each decision tree independently votes for the most possible classification at input *x* [39, 40]. It is a highly flexible and expandable machine learning algorithm based on the concept of integrated learning, which integrates many basic decision tree units into a “forest.” Every decision tree can classify a result through its own features (as shown in Figure 4), and the RF assembles the decisions of all these trees and gives the final decision. It is capable of simultaneously handling thousands of input variables without deletion, and the speed of RF calculation is a lot faster than traditional models.

In this study, we showed that RF is a more powerful method of predicting LVR after AMI. Furthermore, due to its flexibility, scalability, and faster calculation speed, RF is promising in the clinical practice of predicting LVR after AMI.

### 4.5. Clinical Implications

Our study built LVR predictive models with machine learning techniques and discovered that the best 3D STE parameters in predicting LVR after AMI is 3D GLS, and the second is 3D GCS. This model is more accurate because (1) it included 15 risk factors that were encountered regularly in clinical practice and (2) in clinical practice, the sample is always a small and imbalanced one. And this model is more rapid for it needs less calculation steps. Though we have not verified the value of this model in clinical practice, because it is still in its early stage, we believe more and more research will transfer the value of this work into clinical application.

Rapid prediction of future LVR in patients with AMI after PCI is instructive for cardiologists to stratify patients, especially for the detection of patients with poor prognosis. These patients need careful treatment plans to avoid relapse, HF deaths, heart transplantation, and to prevent major ventricular arrhythmia. Further research is required to help supplement the clinical benefits of the model and 3D STE.

### 4.6. Limitations

One of the limitations of this study is that the positive and negative proportion was imbalanced (26 LVR patients vs. 109 non-LVR patients), thus affecting the robustness of the machine learning models. Though RF can reduce this effect, a more balanced data set is still required to give a more convincing result. The other limitation is this study only represents the results of an ultrasound machine from one kind of vendor, so it may be less comparable to results from different vendors. The rigor of this work should be demonstrated by using different ultrasound machines with the similar size of samples.

## 5. Conclusions

There are two main conclusions of this study: (1) the machine learning method Random Forest constructs the best model under the circumstances of predicting LVR with 3D STE; and (2) for AMI patients undergoing effective PCI, the 3D STE parameter 3D GLS acquired at 24 hours after the PCI is highly likely to best predict the occurrence of LVR.

## Figures and Tables

**Table 1:** Demographic and clinical characteristics.

	LVR (*n* = 26)	Non-LVR (*n* = 109)	*p*
Age, yrs	56.85 ± 11.80	53.22 ± 7.92	0.044
Male, %	88.5	89.0	NS
Body mass index, kg/ m^2^	25.67 ± 2.65	24.57 ± 2.77	NS
Body surface area, m^2^	1.75 ± 0.18	1.72 ± 0.14	NS
Medical history			
Hypertension (%)	46.2	42.2	NS
Diabetes (%)	42.3	26.6	NS
Smoking (%)	84.6	89.9	NS
Angiographic findings			
Time to reperfusion (h)	12.69 ± 5.33	10.81 ± 5.67	NS
Multivessel disease (%)	46.2	49.5	NS
LAD as the IRA (%)	65.4	57.4	NS
Blood tests findings			
cTnI	11.54 ± 6.77	9.84 ± 8.49	NS
SCr, *μ*mol/l	85.92 ± 19.05	84.69 ± 21.75	NS
Medications during follow-up			
Antiplatelets (%)	100	100	—
ACEI/ARB (%)	100	95.4	NS
*β*-Blockers (%)	100	95.4	NS
Statins (%)	100	100	—

LAD: left anterior descending branch. IRA: infarct-related artery. ACEI: angiotensin-converting enzyme inhibitors. ARB: angiotensin receptor blockers. NS: *p* > 0.05, nonsignificant.

**Table 2:** Echocardiographic characteristics according to the occurrence of LVR.

	LVR (*n* = 26)	Non-LVR (*n* = 109)	*p*
LVEDV (baseline) (ml)	99.68 ± 16.56	105.431 ± 25.07	NS
LVEDV (follow-up) (ml)	126.94 ± 19.77	105.32 ± 25.53	<0.001
LVESV (baseline) (ml)	47.75 ± 9.96	48.98 ± 18.40	NS
LVESV (follow-up) (ml)	62.39 ± 14.12	47.86 ± 18.34	<0.001
LVEF (baseline) (%)	51.70 ± 6.23	54.40 ± 8.55	NS
LVEF (follow-up) (%)	51.43 ± 7.00	55.42 ± 8.79	0.025
LVMI (baseline) (g/m^2^)	79.79 ± 19.00	85.82 ± 9.33	NS
LVMI (follow-up) (g/m^2^)	81.87 ± 19.34	85.54 ± 8.79	NS
3D GLS (%)	−9.90 ± 2.60	−12.99 ± 3.10	**<0.001**
3D GCS (%)	−14.10 ± 7.53	−18.07 ± 19.49	0.071
3D GAS (%)	−19.72 ± 3.84	−21.33 ± 6.28	NS
3D GRS (%)	28.13 ± 7.13	32.29 ± 9.43	**0.037**

LVEDV: left ventricular end-diastolic volume. LVESV: left ventricular end-systolic volume. LVEF: left ventricular ejection fraction. LVMI: left ventricular mass index. 3D GLS: three-dimensional global longitudinal strain. GCS: global circumferential strain. GAS: global area strain. GRS: global radial strain.

**Table 3:** The correlation of factors with LVR occurrence.

Factors	*r*	*p* value
Age	0.174	**0.043**
cTnI	0.134	0.121
LAD	0.064	0.462
Sex	−0.007	0.939
TTP	0.129	0.137
Smoker	−0.066	0.444
Multivessel	−0.027	0.758
3D GLS	0.396	**<0.001**
3D GAS	0.139	0.107
3D GCS	0.179	**0.038**
3D GRS	−0.185	**0.031**
LVMI	−0.106	0.220
Scr	0.036	0.677

TTP: time to perfusion. BMI: body mass index.

**Table 4:** The univariate analysis of LVR predictive factors.

Factors	OR	95% CI	*p* value
3D GLS	1.374	1.176–1.604	**<0.001**
3D GAS	1.047	0.974–1.125	0.214
3D GCS	1.059	0.992–1.131	0.087
3D GRS	0.949	0.903–0.998	**0.040**
Age	1.049	0.998–1.104	0.061
LVMI	0.962	0.925–1.000	**0.047**
Scr	1.003	0.983–1.023	0.789

**Table 5:** Evaluation of constructed models.

Classifier	AUC	Accuracy (%)	Sensitivity (%)	Specificity (%)	F1 score
DT	0.83	85.71	75.00	88.24	0.81
RF	**0.96**	**90.48**	50.00	**94.12**	**0.85**
eXGB	0.90	76.19	50.00	82.35	0.87
K-NN	0.72	83.82	94.64	**94.87**	0.83
GNB	0.60	70.73	44.44	79.49	0.79
LR	0.83	85.37	92.86	92.31	0.83

## Data Availability

The data used to support the findings of this study are included within the supplementary information files.

## Supplementary Materials

Supplementary Materials 1. Tuning: the tuning of parameters in the construction of a Random Forest model. 2. Random forest construction: the coding of the construction of Random Forest. 3. Raw data: raw data of the research, including all the data of all patients used in the statistical analysis.
