# Supplementary figures and images for: A Comparison of Three-Dimensional Speckle Tracking Echocardiography Parameters in Predicting Left Ventricular Remodeling
Source: J Healthc Eng. 2020 Jul 30;2020:8847144. doi: 10.1155/2020/8847144 (PMC7416266; doi:10.1155/2020/8847144)

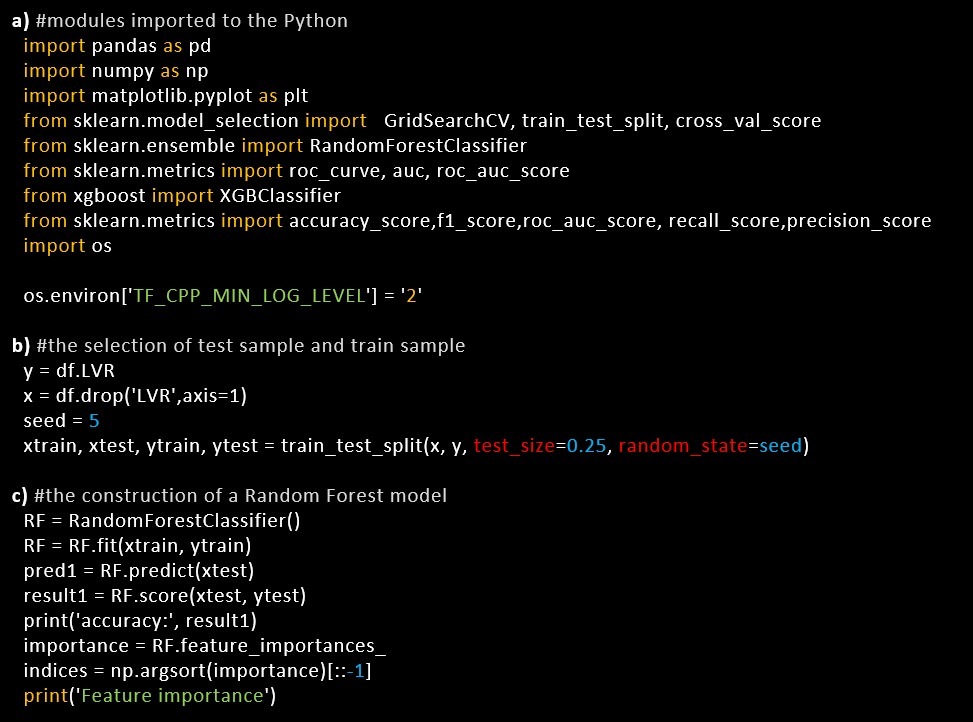

Supplement: Supplementary Materials — 1. Tuning: the tuning of parameters in the construction of a Random Forest model. 2. Random forest construction: the coding of the construction of Random Forest. 3. Raw data: raw data of the research, including all the data of all patients used in the statistical analysis. [file 8847144.f1.zip › 8847144.f1/Random Forest construction.png]

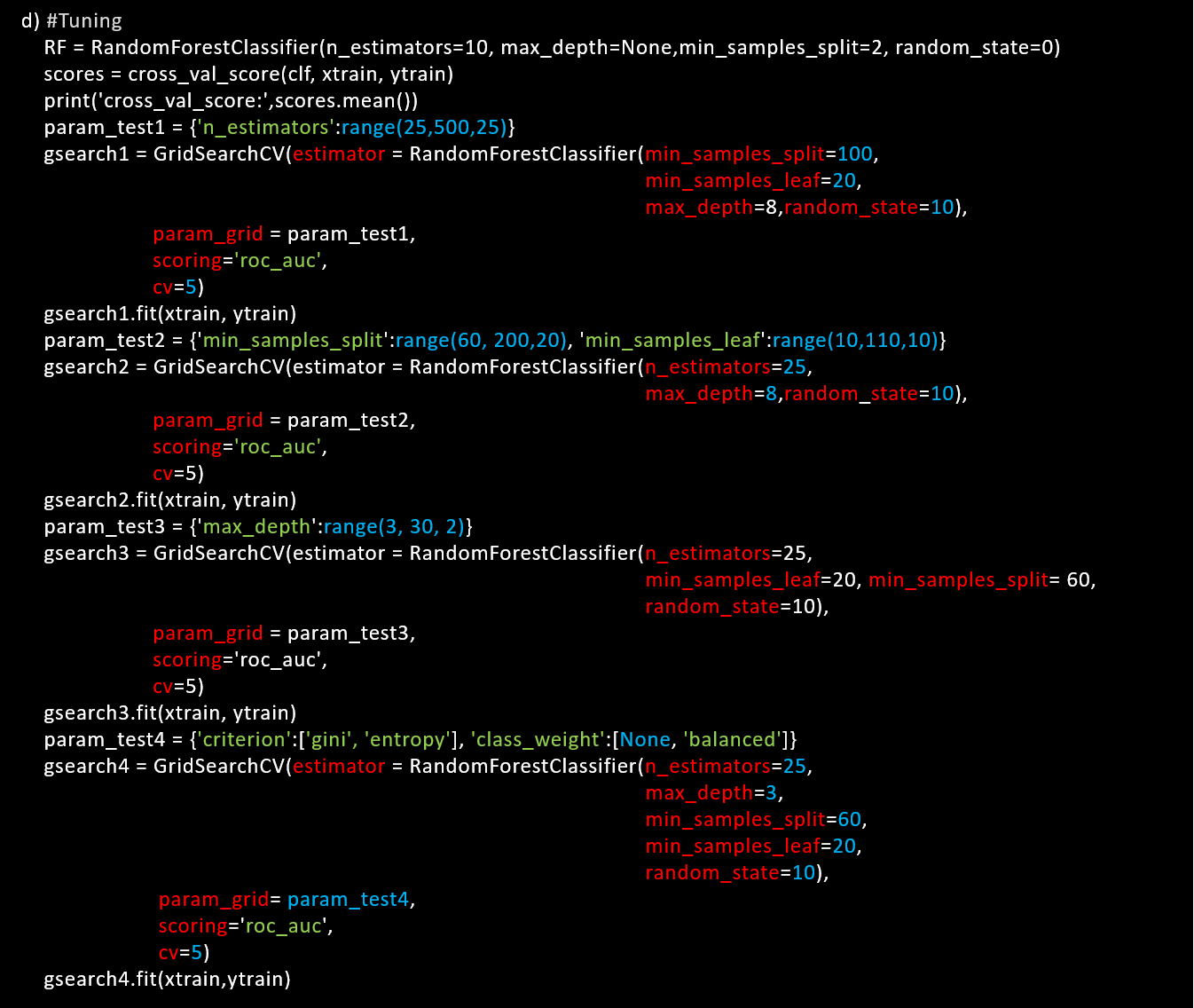

Supplement: Supplementary Materials — 1. Tuning: the tuning of parameters in the construction of a Random Forest model. 2. Random forest construction: the coding of the construction of Random Forest. 3. Raw data: raw data of the research, including all the data of all patients used in the statistical analysis. [file 8847144.f1.zip › 8847144.f1/Tuning.png]
